# Changes in fast food intake in Iranian households during the lockdown period caused by COVID‐19 virus emergency, National Food and Nutrition Surveillance

**DOI:** 10.1002/fsn3.2644

**Published:** 2021-11-01

**Authors:** Samira Rabiei, Delaram Ghodsi, Maryam Amini, Bahareh Nikooyeh, Hamid Rasekhi, Azam Doustmohammadian, Zahra Abdollahi, Mina Minaie, Farzaneh Sadeghi Ghotbabadi, Tirang R. Neyestani

**Affiliations:** ^1^ Department of Nutrition Research National Nutrition and Food Technology Research Institute and Faculty of Nutrition Sciences and Food Technology Shahid Beheshti University of Medical Sciences Tehran Iran; ^2^ Gastrointestinal and Liver Diseases Research Center (GILDRC) Iran University of Medical Sciences Tehran Iran; ^3^ Department of Community Nutrition Deputy of Health Iran Ministry of Health and Medical Education Tehran Iran

**Keywords:** COVID‐19, dietary habits, fast foods, Iran, lockdown

## Abstract

**Background:**

Fast foods, though very popular, are commonly known as unhealthy foods. However, coronavirus pandemic may have influenced on food choices of the general population. This study investigated the changes in fast food consumption during epidemic lockdown 2020.

**Materials and Methods:**

This study was part of a nationwide survey having been conducted using a web‐based electronic self‐administered questionnaire. The questionnaire link was extensively distributed among the whole population both through popular social media platforms and by nutrition and health workers of health offices and medical universities of all provinces. The questionnaire included questions about socio‐demographic characteristics, changes in frequency of fast food consumption as compared with before pandemic, and the reasons for these changes.

**Results:**

A total of 21,290 households took part in the study of whom 89.8% were male‐headed, and almost 74% were from urban areas. Decrease in fast food consumption was reported by 74.8%, while increase in fast food consumption was reported by 2% of the households during the COVID‐19 quarantine. Among those who decreased their fast food consumption, about 82% had completely omitted them from their food baskets. Fear of contamination of fast foods by coronavirus and increase in the number of people at home were the most and the least frequent reasons for decreasing fast food consumption, respectively.

**Conclusion:**

Fast food consumption has dramatically decreased during the lockdown period in Iranian households. Though it may be considered a pleasant offshoot of disastrous COVID‐19 epidemic, the foods replaced fast foods in Iranian households and the overall health consequences warrant further studies.

## Graphical abstract

The result of this study showed that fast food consumption has dramatically decreased during the lock‐down period in Iranian households. Fear of contamination of fast foods by coronavirus and increase in the number of people at home were the most and the less frequent reasons for decreasing fast food consumption, respectively.

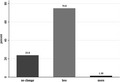

## 1 INTRODUCTION

Coronavirus disease 2019 (COVID‐19), announced as a pandemic by the World Health Organization (WHO), imposed enormous challenges on the economy, health systems, and food supplies globally (Nicola et al., 2020; World Health Organization., 2019). This pandemic has forced many countries to establish partial or total lockdowns to control disease outbreak (Mattioli et al., 2020). These lockdowns have restricted outdoor physical activity and access to fresh foods and have increased mental stress that can severely influence individuals’ lifestyles (Mattioli et al., 2020; Nicola et al., 2020). Indoor activities including working remotely and virtual education, along with restricted outdoor activities and stockpiling food as a result of grocery restriction which imposed by COVID‐19 (Nicola et al., 2020; Sheth, 2020), can affect daily dietary habits and hence the energy intake and may cause a craving for “comfort food” due to boredom and stress (Moynihan et al., 2015; Muscogiuri et al., 2020). Furthermore, the frequent exposure to unsupported claims and news concerning COVID‐19 has a potential to exacerbate the effect of mandatory quarantine on anxiety, fear, and panic (Sidor & Rzymski, 2020). These mental disturbances may occasionally cause over‐eating, in particular high‐calorie and high‐sugar foods (Sinha et al., 2019) in order to cope with the stress, depression, and anxiety and to have a false feeling of happiness (Khubchandani et al., 2020; Rodríguez‐Martín & Meule, 2015; Ruiz‐Roso et al., 2020; Scarmozzino & Visioli, 2020; Sidor & Rzymski, 2020; Zhao et al., 2020). On the other hand, pandemic may result in positive dietary habits, including spending more time for cooking and cutting consumption of unhealthy fast foods (Bennett et al., 2021). Thus, quarantine may have both positive and negative effects on individual's dietary patterns and food choices (Górnicka et al., 2020).

In relation to infectious diseases such as influenza and the new SARS‐CoV‐2 virus, adequate nutrients and healthy nutrition are crucial for supporting the efficient immune system functions (Calder, 2020; Zabetakis & Lordan, 2020). High intake of saturated fatty acids (SFAs), refined carbohydrates, and sweets, as well as low consumption of fibers, unsaturated fats, and antioxidants (Forse & Krishnamurty, 2015; Moravejolahkami et al., 2020) are associated with the high prevalence of type 2 diabetes and obesity which are two prominent risk factors for developing severe forms of COVID‐19 (Richardson et al., 2020). Fast foods typically contain high amounts of fat and industrially produced trans‐fatty acids (TFAs) (Vaughan et al., 2019). The association between diet high in fat including TFAs and the increased risk of weight gain, dyslipidemia, and consequent stimulation of the inflammatory reactions has already been observed in experimental as well as human studies (Bendsen et al., 2011a; Bendsen et al., 2011b; Kavanagh et al., 2007). Furthermore, industrial TFAs have been associated with increased asthma risk and lung inflammation (Wood et al., 2011) and can indirectly worsen COVID‐19 manifestations especially respiratory complications (Bohlouli et al., 2021). There is inconsistency in the results of studies regarding fast foods consumption during COVID‐19 self‐confinement. While some studies reported a significant reduction in the frequency of the consumption of fast foods, instant soups, or ready‐made jars during pandemic (Abouzid et al., 2021; Błaszczyk‐Bębenek et al., 2020), some others reported an increase in fast food consumption (Ben Hassen et al., 2020; Górnicka et al., 2020). The impact of the pandemic on dietary behaviors and food choices could, therefore, differ in various populations. In this nationwide study, we aimed to evaluate changes in the frequency of fast foods consumption in Iranian households during lockdown period as compared with before pandemic. We also evaluated the reasons behind these changes in a representative sample of the whole country.

## 2 MATERIAL AND METHODS

### 2.1 Study design

This study was part of a nationwide cross‐sectional descriptive–analytical survey having been conducted using a web‐based electronic self‐administered questionnaire. The questionnaire was completed from 4 to 25 April, 2020, during which Iran was in coronavirus epidemic lockdown. The questionnaire used in the study was developed by an expert panel through several virtual sessions aiming at evaluation of any changes in the dietary pattern including fast foods intake among the Iranian households during coronavirus quarantine. The content validity was assured by an expert panel of seven internal (inside NNFTRI) and three external (from other institutions) nutritionists. The questionnaire was uploaded via a web link (https://digit.rabit.info/s/2ZRaVKMB.html). An official letter was sent from the Community Nutrition Department of Ministry of Health to the vice‐chancellors in health affairs and the Community Nutrition Offices of the medical universities of all provinces. Through this letter, the objectives of the study were explained and they were requested to share the link through provincial health and nutrition workers among the community under their service coverage. Furthermore, the link was distributed massively to popular social media platforms such as Telegram and WhatsApp. The protocol of the study and the details of the questionnaire have been described elsewhere (Rasekhi et al., 2021).

### 2.2 Data collection

The web‐based questionnaire contained 2 sections: (a) questions on socio‐economic status of the households including data regarding gender, education and occupation of the head of the household, household size, residency area (urban/rural), presence of high‐risk person in the household (under‐5 children, pregnant or lactating women, elder, presence of a person with the history of COVID‐19), and any changes in household income during the coronavirus epidemic; and (b) changes in frequency in consumption of fast foods (in addition to the other food groups) and the reasons for changes of consumption pattern during the pandemic. Respondents who were one member of each household aged more than 18 years were asked to complete the questionnaire on behalf of his/her household. A brief description of the study, its objectives, and a declaration of anonymity and confidentiality were given to the participants before starting the questionnaire to assure the participants of privacy of their responses. The answers were saved only by clicking the submit button after completing the questionnaire.

### 2.3 Statistical analysis

All data were analyzed using Stata version 16.0 (StataCorp LLC). Descriptive statistics were calculated for all variables where categorical variables were presented as frequency and percentage. To compare variables among provinces with different food security situations, the latest national report was used in which provinces are categorized as food insecure (deprived), semi‐secure (semi‐deprived), and secure (non‐deprived) (Kolahdooz & Najafi, 2012). Ordinal logistic regressions were applied to examine which factors contributed to changes in frequency of consumption of fast food. A 2‐tailed *p* < .05 was considered significant. Two outcomes were considered as dependent variables in regression models: 1. Changes in frequency of fast food consumption on a weekly basis (increase vs. no changes vs. decrease) 2. Decrease in consumption of fast food (small decrease vs. half vs. omitted) after the test for overall parallel assumption at 0.05 significance indicated that the overall parallel assumption has not been violated (*p* = .515 and .133).

The sex of household head (male and female), residency area (urban/rural), household size (one to two, three to five, six, and more), being high‐risk member(s) in a household (none, least one child under five, pregnant/ lactating, elder, and more than one member), household head's occupation (employee, self‐employee, retired, health worker, teacher, driver, and other) and educational (master and higher, bachelor, associate, diploma, and high school), changes in income (no changes, small decrease, half, and cut), COVID‐19 in family (no and yes), and food security status of the province (secure, semi‐secure, and deprived) were the independent variables assessed. In all analyses, sampling weights were used to account for the complex sampling design and to allow inferences valid for the population.

## 3 RESULTS

A total of 21,290 households from all provinces of the country completed the questionnaire and were included in the study. The mean age of household's head was 44.7 (95% confidence interval [CI]; 44.2– 44.9). The household size of over 80% of the participants was 3 or more (Table 1). The mean household size and the ratio of urban to rural households are in good agreement with the report from latest population census in Iran (Iran Statistics Center, 2019), suggesting that the study population was representative (~1%) of the whole country households.

**TABLE 1:** Socio‐economic characteristics of the participants (*n* = 21,290), April 2020

Variables		*n* (%)^a^
Sex of head	Male	19,255 (89.8)
	Female	2035 (10.2)
Urban/Rural	Urban	14,191 (73.8)
	Rural	7099 (26.2)
Household size	1–2	2883 (15.7)
	3–5	16,798 (78.2)
	>6	1609 (6.1)
High‐risk members in household	None	11,511 (54.1)
	Under five years old	4881 (22.9)
	Pregnant/ lactation	660 (3.1)
	Elder	2110 (3.9)
	More than one member	2128 (23.8)
Occupation	Officer	3942 (20.5)
	Unemployed	7755 (34.3)
	Retired	1988 (11.7)
	Health workers	572 (2.7)
	Teacher	715 (3.1)
	Driver	883 (3.9)
	Other	5435 (23.8)
	No diploma (No high school completion)	8113 (33.0)
	Diploma	5277 (24.1)
	Associate	1540 (7.3)
	Bachelor	4022 (21.2)
	Master/higher	2338 (14.4)

^a^ Percentages are weighted.

The most and the least frequent types of occupation stated were self‐employed (34.3%) and health worker (2.7%), respectively. As for education level of household's head, under diploma and associate degree had the most (33%) and the least (7.3%) frequency, respectively.

**FIGURE 1:**
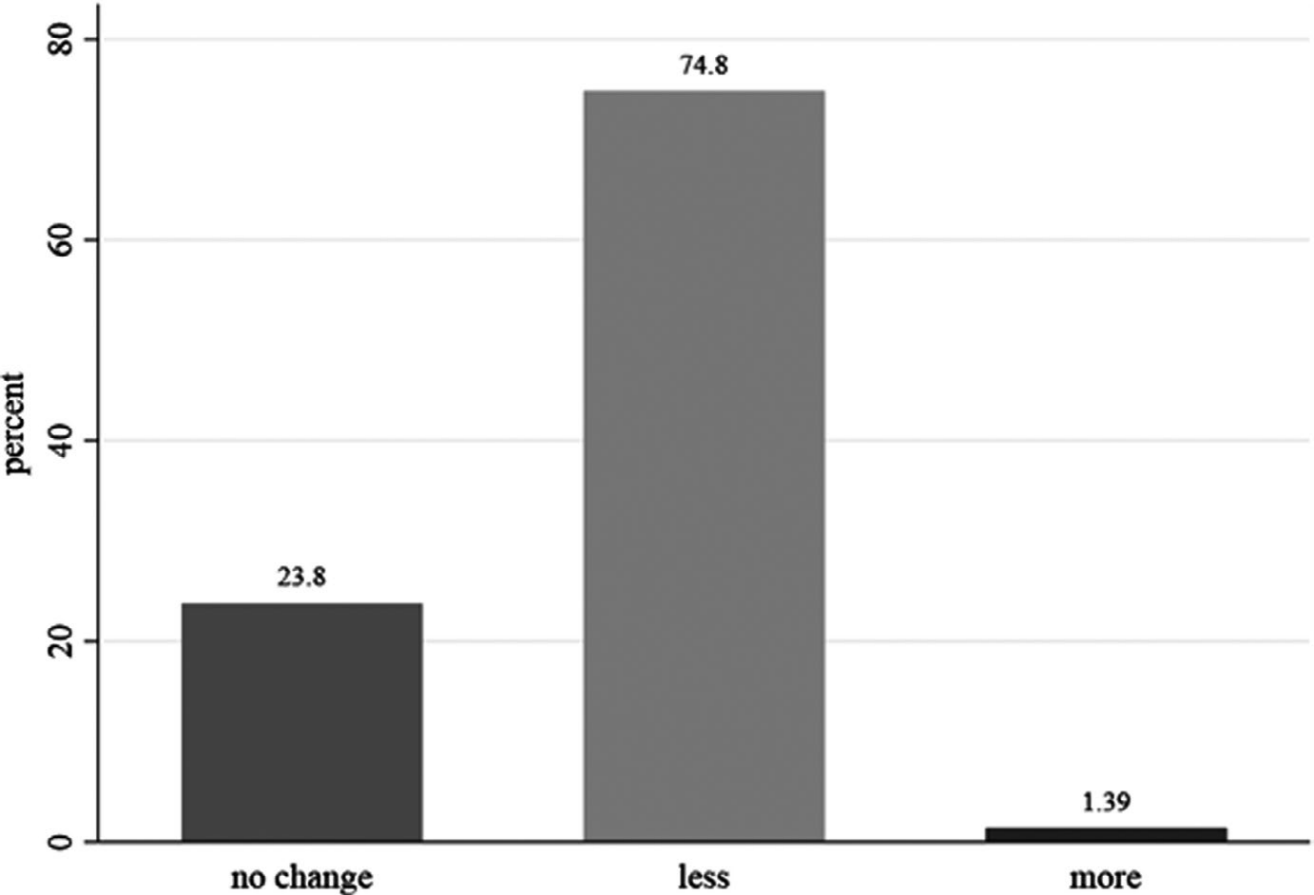
Changes in weekly consumption of fast foods in household during COVID‐19 quarantine

A remarkable change in frequency of fast foods consumption during COVID‐19 quarantine was reported by about 76.2% of the participants, of whom 74.8% had decreased and only 2% had increased fast foods consumption (Figure 1).

Ordered logistic regression was employed to analyze statistically significant predictors of change in fast foods consumption during COVID‐19 quarantine among the respondent households (Table 2). The dependent variables were changes in frequency of fast foods consumption following COVID‐19 epidemic in the models (increased consumption, no changes, or decreased consumption). The results showed that the female‐headed households, as compared with male‐headed ones, were 22% more likely to decrease their weekly consumption of fast foods during COVID‐19 quarantine (OR = 1.22, 95% CI: 1.04, 1.42, *p* = .013). Area of residency was the other predictor of the intake of fast foods. According to this model, households resided in rural areas were almost 50% less likely to decrease their fast foods consumption during the epidemic (OR = 0.48, 95% CI: 0.44, 0.53; *p* < .001).

**TABLE 2:** Ordered Logistic Regression Models of changes in weekly consumption intake of fast foods during COVID‐19 quarantine

Variables	Fast foods		
	Odds ratio	(95% CI)	*p*‐value
Sex of household head			
Male	—	—	—
Female	1.22	1.04, 1.42	.013
Urban/Rural			
Urban	—	—	—
Rural	0.48	0.44, 0.53	<.001
Household size			
1–2	—	—	—
3–5	1.43	1.26, 1.62	<.001
>6	1.66	1.40, 1.97	<.001
High‐risk members			
No	—	—	—
<5 years old	0.89	0.80, 0.98	.031
Pregnant/lactating	1.27	0.99, 1.62	.057
Women	0.99	0.86, 1.15	.960
Elder	0.97	0.84, 1.13	.749
More than one			
Occupation			
Employee	—	—	—
Self‐employee	0.60	0.51, 0.71	<.001
Retired	0.85	0.70, 1.05	.138
Health workers	0.89	0.64, 1.24	.512
Teacher	0.83	0.62, 1.11	.231
Driver	0.61	0.47, 0.79	<.001
Other	0.51	0.43, 0.60	<.001
Change in income			
No changes	—	—	—
Low decrease	3.30	2.86, 3.81	<.001
Half	2.79	2.46, 3.18	<.001
Cut	3.85	3.35, 4.43	<.001
Covid−19 in household			
No	—	—	—
Yes	0.80	0.63, 1.02	.076
Education			
Master/ higher	—	—	—
Bachelor	0.89	0.73, 1.09	.287
Associate	0.90	0.71, 1.14	.410
Diploma	0.79	0.64, 0.97	.029
High school	0.58	0.47, 0.72	<.001
Food Security status of province			
Secure	—	—	—
Semi‐secure	1.03	0.91, 1.17	.576
Deprived	0.83	0.72, 0.94	.006

Household size was a determinant as the households with 3–5 members, and those with more than 6 members were more likely to decrease their weekly fast foods consumption as compared with households with 1–2 members (OR = 1.43, 95% CI: 1.26–1.62, and OR=1.66, 95% CI: 1.40–1.97; *p* < .001, respectively). Households who had at least one under‐5 child were 11% less likely to decrease their weekly fast food consumption compared with the households with no high‐risk person (OR = 0.89, 95% CI: 0.8–0.98; *p* < .03). Households whose income was changed during COVID‐19 epidemic, as compared with those with no change in income during this period, were more likely to decrease the frequency of their fast food consumption. Households with lower household's head educational level were 42% less likely to decrease their weekly fast food consumption during the COVID‐19 epidemic compared with those with higher level of education. Interestingly, the households who lived in deprived provinces were 17% less likely to decrease their weekly fast foods consumption as compared with those who lived in food secure provinces (OR = 0.83, 0.95% CI (0.72–094; *p* < .001)).

**FIGURE 2:**
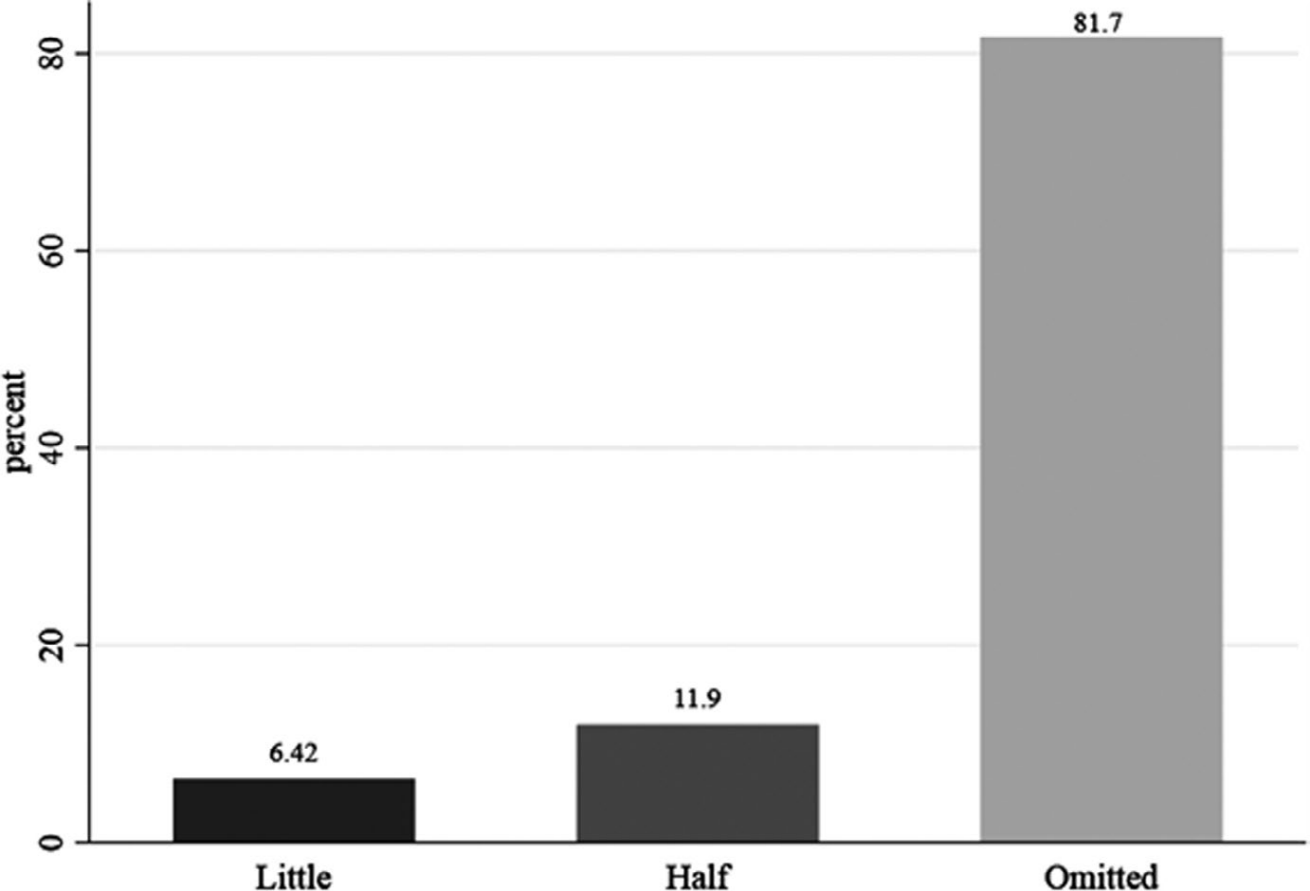
Decrease in weekly fast foods consumption among the households reported decreasing during COVID‐19 quarantine

Among the households who reported a reduction in weekly consumption of fast foods, about 82% completely had omitted these foods from their baskets (Figure 2).

The results of ordered regression to identify predictors of reduction in weekly fast foods consumption during COVID‐19 quarantine are shown in Table 3. The analysis revealed that living in rural areas was associated with less reduction in weekly consumption of fast foods. Households who had a high‐risk member, except for pregnant/lactating women, were more likely to omit their weekly fast foods consumption. Furthermore, households whose income was cut during COVID‐19 quarantine were more likely to omit their weekly fast foods consumption. Interestingly, households from deprived provinces were 25% less likely to omit their weekly fast foods intake compared with those residing in secure provinces.

**TABLE 3:** Ordered Logistic Regression Models of decease in weekly intake of fast foods during COVID‐19 quarantine

Variables	Fast foods		
	Odds ratio	(95% CI)	*p*‐value
Sex of household head			
Male	—	—	—
Female	0.85	0.71, 1.02	.093
Urban/Rural			
Urban	—	—	—
Rural	0.83	0.73, 0.94	.005
Family members			
1–2	—	—	—
3–5	0.77	0.64, 0.92	.005
>6	0.71	0.56, 0.91	.008
High‐risk members			
No	—	—	—
<5 years old	1.25	1.08, 1.43	.002
Pregnant/lactating women	1.17	0.86, 1.60	.304
Elder	1.30	1.04, 1.63	.017
More than one	1.50	1.25, 1.82	<.001
Occupation			
Employee	—	—	—
Self‐employee	0.89	0.73, 1.08	.256
Retired	1.32	1.02, 1.70	.030
Health workers	0.97	0.69, 1.37	.882
Teacher	1.04	0.78, 1.38	.775
Driver	1.22	0.91, 1.64	.179
Other	1.08	0.88, 1.32	.426
Change in income			
No changes	—	—	—
Low decrease	1.01	0.86, 1.18	.876
Half	1.08	0.91, 1.28	.365
Cut	1.48	1.23, 1.78	<.001
COVID−19 in household			
No	—	—	—
Yes	0.92	0.69, 1.23	.598
Education			
Master./ higher	—	—	—
Bachelor	1.06	0.87, 1.30	.521
Associate	1.0	0.77, 1.29	.975
Diploma	1.16	0.93, 1.44	.178
High school	0.99	0.78, 1.24	.938
Security status of province			
Secure	—	—	—
Semi‐secure	1.02	0.89, 1.17	.724
Deprived	0.75	0.64, 0.88	.001

**FIGURE 3:**
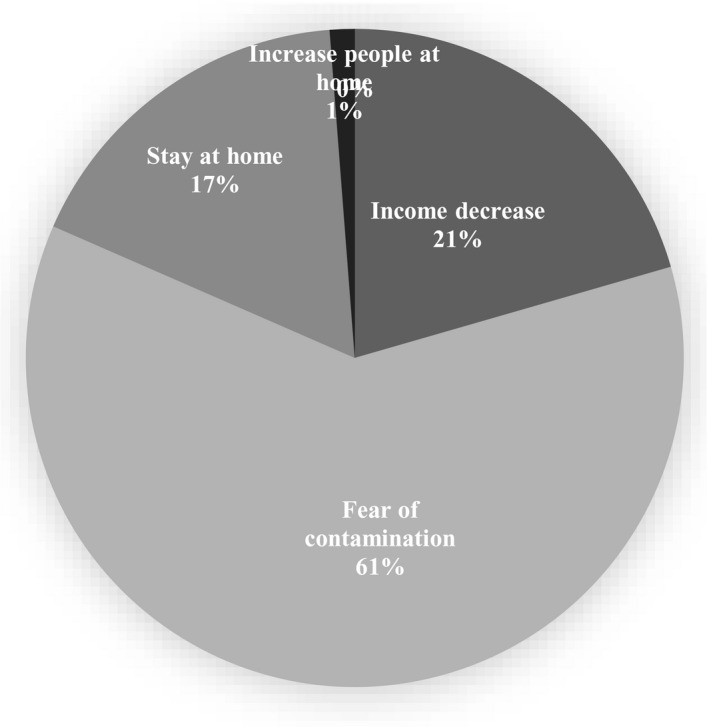
Reasons of decrease in household's weekly fast food consumption during COVID‐19 quarantine

Fear of contamination of fast foods by coronavirus (~60%) and increase in the number of people present at home at meal times (1%) were the most and the least frequent reasons for decreasing fast foods consumption during this period, respectively (Figure 3).

**FIGURE 4:**
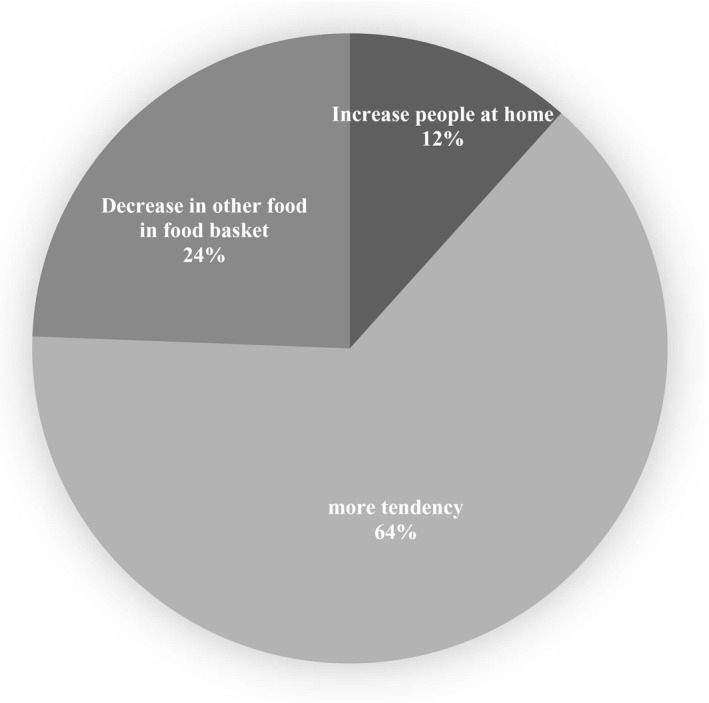
FIGURE Reasons for increase in weekly fast food intake during COVID‐19 quarantine

Within households who increased their intake of fast foods, more inclination for fast foods consumption was the main reason. Decrease in consumption of other food items in the household food basket and increase in the number of individuals at meal time were reported by 24% and 12% of households as the other reasons for increasing in fast foods intake during the epidemic, respectively (Figure 4).

## 4 DISCUSSION

Our study showed that in more than half of the households, the frequency of fast foods consumption was remarkably decreased, and even in some households, fast foods were totally excluded from their baskets during COVID‐19 quarantine restrictions as compared with before lockdown period.

Our findings are in accord with a recent study conducted in 17 countries of the Middle East and North Africa regions, which showed the percent of fast food non‐consumers dramatically increased during coronavirus epidemic (48.8%) as compared with before this period (25.4%). Furthermore, decrease in fast foods consumption was significantly more among females than in males (Abouzid et al., 2021). Interestingly, we also found that female‐headed households had more tendency to decrease their fast foods consumption than male‐headed ones during COVID‐19 epidemic. However, the reasons behind these changes could be different in our study. Female‐headed households might have been more economically under pressure whereby had less purchasing power during lockdown period (Shahzad et al., 2021). Alternatively, in female‐headed households woman, as the main person responsible for both buying and preparing food might more consider other food choices when there is less tendency to have fast foods for any reason. Błaszczyk‐Bębenek et al. also showed a significant increase in consumption of eggs, potatoes, sweets, canned meat, and alcohol and a significant decrease in fast food products, instant soups, and energy drinks consumption during lockdown (Błaszczyk‐Bębenek et al., 2020). A decrease in total fast foods consumption during lockdown has also been reported in Kuwait, Spain, Italy, Brazil, Colombia, and Chile (Husain & Ashkanani, 2020; Ruiz‐Roso et al., 2020). Significant reduction of eating out and ordering food during lockdown may be among the reasons for decreasing fast foods intake (Błaszczyk‐Bębenek et al., 2020).

Although McGuire et al. showed an increase in the use of food delivery systems during lockdown period (McGuire, 2011), the results of a systematic review showed a significant decrease in fast food consumption during the lockdown period in seven of eight investigated studies (Cheikh Ismail et al., 2020; Górnicka et al., 2020; Husain & Ashkanani, 2020; Kriaucioniene et al., 2020; Rodríguez‐Pérez et al., 2020; Rolland et al., 2020). Tendency to eat healthier foods in response to the spread of COVID‐19 (Husain & Ashkanani, 2020) and staying at home and having more free time for preparing homemade food may have resulted in reducing fast foods consumption, as already stated in other studies (Di Renzo et al., 2020; Górnicka et al., 2020; Sidor & Rzymski, 2020). Spending more time for cooking and reduced fast food intake may be considered as the positive lifestyle habits emerging from the pandemic (Bennett et al., 2021). It is noteworthy that closing fast food outlets, public meetings ban, closed restaurants with only take‐away meals, or door‐to‐door food deliveries during lockdown have significantly affected fast food intake (Husain & Ashkanani, 2020; Zabdyr‐Jamróz & Kowalska‐Bobko, 2020). Another explanation for decreasing fast foods during COVID‐19 pandemic which is currently not proven and needs further research could be the fear of transmission of COVID‐19 disease via packets of food and delivery driver (Ben Hassen et al., 2020). This issue was also mentioned by the majority of our respondents as the main reason for decreasing their fast food intake during the epidemic lockdown.

On the other hand, we found an increase in fast foods consumption in 1.39% of households. Among them, 64% reported an increase in fast foods consumption due to more inclination for these kinds of foods. Increased consumption of fast foods has also been reported in Canada (Zajacova et al., 2020) and Poland (Górnicka et al., 2020). It has been stated that anxiety, boredom, and emotional and psychological response to quarantine could result in drastic changes in lifestyle including provocation for over‐eating, less care about diet quality, and decreased physical activity (Ben Hassen et al., 2020; Naja & Hamadeh, 2020). It can, therefore, be challenging to maintain healthy food habits and regular physical activity during lockdown (Montemurro, 2020; Wu et al., 2020). Considering the pre‐pandemic tremendous increase in fast foods consumption (Hilary & Lauren, 2011; Niemeier et al., 2006) and its potential role in development of several non‐communicable diseases such as obesity and the related comorbidities including aggravation of COVID‐19 severity (Block et al., 2004; Calder, 2020; Di Renzo et al., 2020; Hauner, 2005), the decrease in fast foods consumption may seem a pleasant offshoot of the disastrous pandemic. However, it may be just an over‐simplistic notion as this is not the only change in the people's dietary pattern happened due to the pandemic. The quantity and quality of the foods replaced fast foods are among the determinants of the final health outcomes of these dietary changes.

In our study, households with at least one under‐5 child were less likely to decrease fast foods intake, whereas households whose income had dramatically decreased during the quarantine and who lived in deprived provinces were more likely to decrease or omit the fast foods from their baskets. On oppose to our findings, in a study in Poland, the households with children and those living in a region with higher Gross Domestic Product (GDP) had higher chance of adherence to an unhealthy dietary pattern (Górnicka et al., 2020). It has been shown that job and income losses related to government‐mandated shutdowns resulted in adverse effects on food security of vulnerable people in low‐ and middle‐income countries. The poor will respond by spending the majority of income on foods and purchasing the cheapest calories like fast foods which generally are rich in calories, fat, and sugar but are poor in nutrients (Headey & Ruel, 2020). On the contrary, we found income losses were associated with a decrease in fast foods consumption. It is worth to note that COVID‐19 has influenced food choices in our studied population.

Generally, since the COVID‐19 has no effective preventive and pharmacological therapies available at the moment, maintaining healthy dietary behavior and life style may be beneficial to improve immune responses and prevent viral infections, especially for vulnerable population (Di Renzo et al., 2020). The interesting findings are that COVID‐19 pandemic, apart from its all disastrous aspects, has provided an unexpected opportunity for healthy eating. Though decreasing unhealthy fast foods intake can certainly be beneficial for health but the substitutes for these popular foods are also equally important. This issue warrants further studies.

## 5 CONCLUSION

Our results revealed decreasing consumption of fast foods in most of the studied households during quarantine. Changes in dietary behaviors and regular lifestyle induced by quarantine have important effects on health, especially the strength of immune system. The impact of these changes is not limited to quarantine period. The consequents of these changes can influence public health not only during the pandemic, but also after this period. Although fast foods consumption has decreased during the quarantine, appropriate public health interventions are needed to maintain this decreasing trend not only during epidemic, but also after this period. Further studies to evaluate multi‐aspects of changes in fast food consumption and their consequences are warranted.

## 6 STRENGTH AND LIMITATIONS

To our knowledge, this was the first study to investigate the immediate impact of the COVID‐19 pandemic on dietary pattern of Iranian population. Using an online survey as an ideal research tool during the lockdown allowed recruiting a large sample from different regions of the country. One of the strengths of this study is the collection of large data from all over Iran during the critical time, which can suggest public health policies for people in the nationwide. However, some limitations should be acknowledged. One of the main limitations of this web survey is self‐reported data, without any verification by other methods such as focused interviews and food diaries. Furthermore, the perception of household members of "changes in intake frequency" might be different. It could influence the reliability of the collected data and could be prone to bias and misreporting. However, we tried to minimize this problem by designing very clear and easy to reply questions and also the web‐based application as much user‐friendly as possible. The other limitation of our study was the qualitative nature of our data. In other words, we did not have any information about the amount of fast food consumption before and during epidemic.

## AUTHOR CONTRIBUTIONS

**Samira Rabiei:** Conceptualization (equal); Investigation (equal); Methodology (equal); Validation (equal); Writing‐original draft (equal); Writing‐review & editing (equal). **Delaram Ghodsi:** Conceptualization (equal); Investigation (equal); Methodology (equal); Validation (equal); Writing‐original draft (equal); Writing‐review & editing (equal). **Maryam Amini:** Conceptualization (equal); Investigation (equal); Methodology (equal); Validation (equal). **Bahareh Nikooyeh:** Conceptualization (equal); Data curation (equal); Formal analysis (equal); Investigation (equal); Methodology (equal); Validation (equal). **Hamid Rasekhi:** Conceptualization (equal); Data curation (equal); Investigation (equal); Methodology (equal); Software (equal); Validation (equal). **Azam DoustMohammadian:** Conceptualization (equal); Investigation (equal); Methodology (equal); Validation (equal). **Zahra Abdollahi:** Conceptualization (equal); Supervision (equal); Validation (equal). **Mina Minaie:** Conceptualization (equal); Supervision (equal); Validation (equal). **Farzaneh Sadeghi Ghotbabadi:** Conceptualization (equal); Supervision (equal); Validation (equal). **Tirang‐Reza Neyestani:** Conceptualization (equal); Investigation (equal); Methodology (equal); Project administration (leader); Supervision (leader); Validation (equal); Writing‐review & editing (equal).

## ETHICAL STATEMENT

All procedures were approved by Ethics Committee of the National Nutrition and Food Technology Research Institute (IR.SBMU.NNFTRI.REC.1399.066).

## Data Availability

Data described in this manuscript will be made available upon request to the project's leader.
